# Some (bacilli) like it hot: genomics of Geobacillus species

**DOI:** 10.1111/1751-7915.12161

**Published:** 2014-09-05

**Authors:** David J Studholme

**Affiliations:** Biosciences, University of ExeterGeoffrey Pope Building, Stocker Road, Exeter, EX4 4QD, UK

## What are *Geobacillus*?

The genus *Geobacillus* includes thermophilic Gram-positive spore-forming bacteria that form a phylogenetically coherent clade within the family *Bacillaceae*. They are of great interest for biotechnology (as discussed below). These thermophiles seem to be ubiquitous; viable *Geobacillus* spores can be isolated in large quantities not only from hot environments such as hydrothermal vents, but also, paradoxically, from cool soils and cold ocean sediments (Zeigler, 2005).

**Fig 1:**
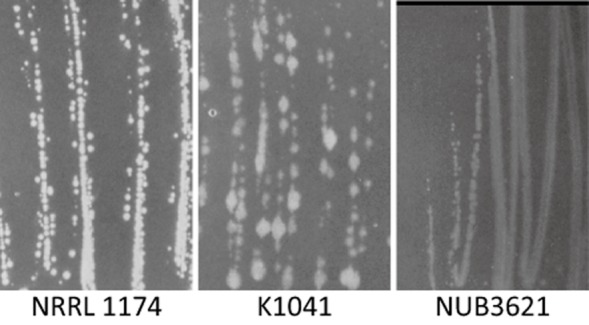
Diverse colony morphologies of strains classified as ‘*G. stearothermophilus*’. Strains NRRL 1174, K1041 and NUB3621 were streaked-out on tryptic soy broth plates and incubated overnight at 50°C. Plates were photographed under identical conditions.

These bacteria were previously categorized as ‘Group 5’ within the genus *Bacillus* but were subsequently split into the new genus *Geobacillus* (Nazina *et al*., 2001). Many *Geobacillus* strains were previously described as belonging to a single species *Bacillus stearothermophilus*, but it was clear that there was great heterogeneity in physiology, preferred temperature range and other phenotypic characteristics among these strains. For example, see Fig. 1 showing three distinct colony morphologies among three strains described as ‘*B. stearothermophilus*’. It is now absolutely clear that there are several distinct species within *Geobacillus* and these can be distinguished by both genotype and phenotype (Nazina *et al*., 2001; Banat *et al*., 2004; Zeigler, 2005; Dinsdale *et al*., 2011; Coorevits *et al*., 2012).

## Why are *Geobacillus* species of interest for biotechnology?

*Geobacillus* spp. are of interest for biotechnology as source of thermostable enzymes and natural products, digesters of lignocellulose, bioremediators of hydrocarbons, producers of bio-fuel, cellular factories for heterologous expression of enzymes and as hosts for directed evolution (Wiegel *et al*., 1985; Niehaus *et al*., 1999; Couñago and Shamoo, 2005; Marchant *et al*., 2006; Cripps *et al*., 2009; Taylor *et al*., 2009; Tabachnikov and Shoham, 2013). Industrially important enzymes originating from *Geobacillus* spp. include lipases (Schmidt-Dannert *et al*., 1998), glycoside hydrolases (Fridjonsson *et al*., 1999; Bartosiak-Jentys *et al*., 2013; Suzuki *et al*., 2013), N-acylhomoserine lactonase (Seo *et al*., 2011) and DNA polymerase I (Sandalli *et al*., 2009) and protease (Chen *et al*., 2004) among others. The advantages of using thermophilic bacteria as whole-cell biocatalysts were recently discussed in this journal (Taylor *et al*., 2011) and include reduced risk of contamination, acceleration of biochemical processes and easier maintenance of anaerobic conditions. These bacteria also tend to ferment a wide range of substrates, utilizing both cellobiose and pentose sugars. In the context of bioethanol production, there is the additional advantage of reduced cooling costs and easier removal and recovery of the volatile product by sparging or partial vacuum thus also avoiding ethanol poisoning of the bacteria (Taylor *et al*., 2009). Less positively, *Geobacillus* spp. are common contaminants in the dairy and food industries (Burgess *et al*., 2010).

## Which genomes have been sequenced?

**Fig 2:**
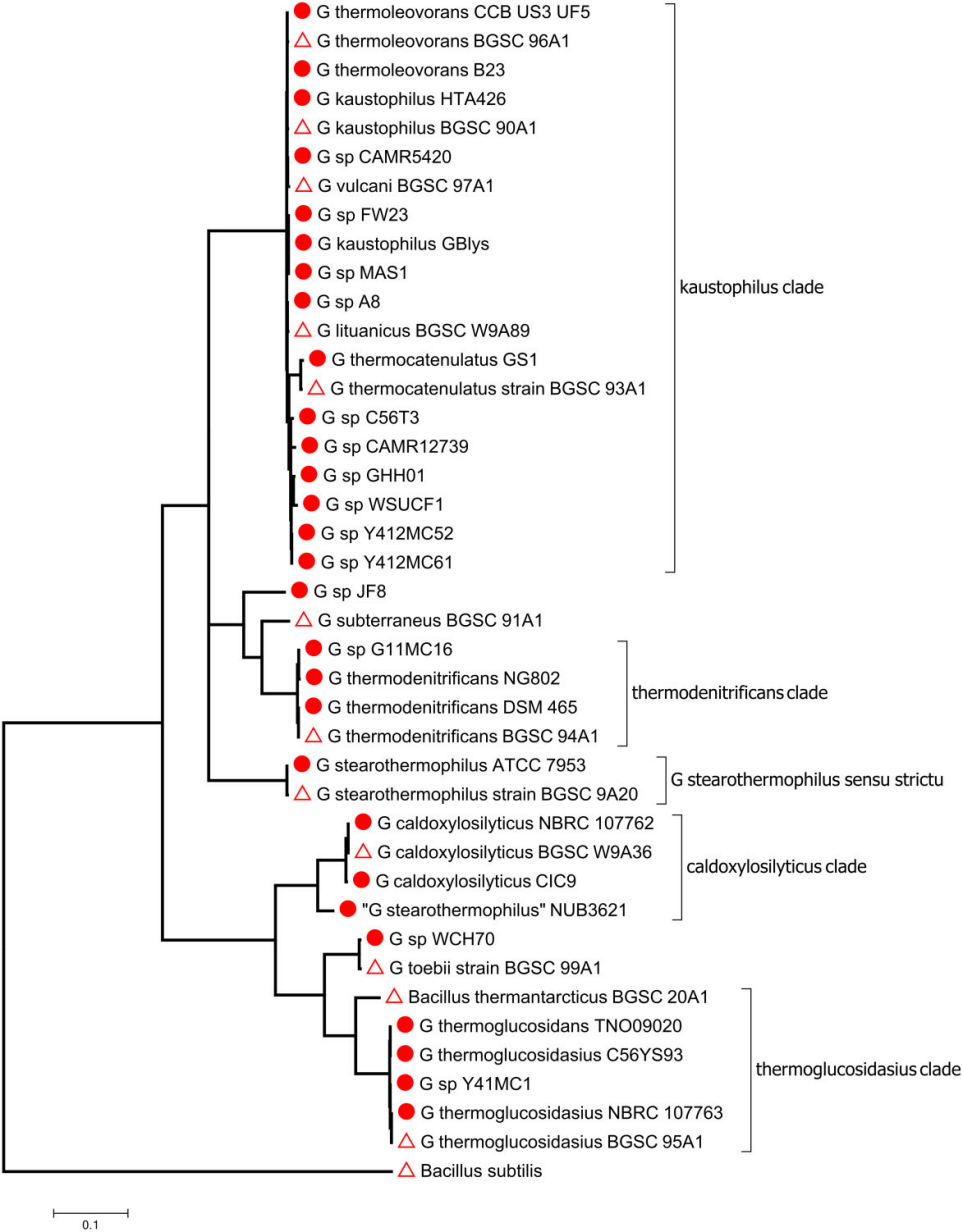
Phylogenetic relationships among sequenced strains of *Geobacillus* inferred from a multiple sequence alignment of *recN* sequences. The circles indicate strains whose genomes have been sequenced, as listed in Table 1. The triangles indicate type strains of the various *Geobacillus* species; *recN* sequences from these are taken from a previous phylogenetic analysis by Zeigler (2005). The maximum-likelihood tree was generated using mega6 (Tamura *et al*., 2013).

At the time of writing (28 July 2014), 29 *Geobacillus* genome sequences are available (Table 1). These include representatives of all the major phylogenetic groups within the genus and include representatives of the species *G. thermoleovorans*, *G. kaustophilus*, *G. thermocatenulatus*, *G. thermodenitrificans*, *G. stearothermophilus*, *G. caloxylosilyticus* and *G. thermoglucosidans* (formerly *G. thermoglucosidasius*) as well as several strains that have not been assigned to named species (Fig. 2). Genome sequences are also available for some other thermophilic members of the *Bacillaceae*, such as *Paenibacillus lautus* (Mead *et al*., 2012) and *Bacillus coagulans* (Xu *et al*., 2013) and for *Geobacillus*-infecting bacteriophage (Marks and Hamilton, 2014), but these will not be discussed here. The team who sequenced the genome of *Geobacillus* sp. MAS1 described this strain as ‘*G. thermopakistaniensis*’, but this is not a validly named species and no justification was provided for its proposal as a new species (Siddiqui *et al*., 2014). On the basis of its *recN* sequence, a useful phylogenetic marker for *Geobacillus* spp. (Zeigler, 2005), strain MAS1 is closely related to the type strains of *G. kaustophilus* and *G. thermoleovorans* (Fig. 2). Strain NUB3621 was described as ‘*G. stearothermophilus*’ but as has been previously noted (Studholme *et al*., 1999; Zeigler, 2005; Blanchard *et al*., 2014), this strain is phylogenetically distinct from *B. stearothermophilus sensu strictu* and is more closely related to *G. caldoxylsilyticus* and, to a lesser extent, *G. thermoglucosidans* (Fig. 2). For more than half of the sequenced genomes, papers have been published describing and/or announcing the sequence data and usually indicating the particular features of the strain that motivated its sequencing. An insightful discussion of the biological lessons from *Geobacillus* genomes was previously published earlier this year, including surveys of genes involved in breakdown of plant-derived lignocellulose (Zeigler, 2005); but at that time, only 10 genome sequences were available.

**Table 1:** *Geobacillus* strains whose genomes have been sequenced as of 26 July 2014

Species and strain	Motivation for sequencing	Accession number	References
*G. caldoxylosilyticus* CIC9	Not known	NZ_AMRO01000000.1	n. a.
*G. caldoxylosilyticus* NBRC 107762	Not known	BAWO01000000.1	n. a.
*G. kaustophilus* GBlys	Lysogenic, containing an integrated prophage	NZ_BASG01000001.1	(Doi *et al*., 2013)
*G. kaustophilus* HTA426	Source of novel glycoside hydrolases (6-phospho-β-glycosidase and β-fucosidase)	NC_006510.1	(Takami *et al*., 2004)
*G.* sp. A8	Not known	NZ_AUXP01000001.1	n. a.
*G.* sp. C56-T3	Not known	NC_014206.1	n. a.
*G.* sp. CAMR12739	Hemicellulose degradation	JHUR01000001.1	(De Maayer *et al*., 2014)
*G.* sp. CAMR5420	Hemicellulose degradation	JHUS01000001.1	(De Maayer *et al*., 2014)
*G.* sp. FW23	Potential for degradation and utilization of oil (bioremediation of oil spills)	JGCJ01000001.1	(Pore *et al*., 2014)
*G.* sp. G11MC16	Not known	NZ_ABVH01000001.1	n. a.
*G.* sp. GHH01	Source if thermostable and thermo-active secreted lipase	NC_020210.1	(Wiegand *et al*., 2013)
*G.* sp. JF8	Degrades biphenyl and polychlorinated biphenyls (PCB)	NC_022080.4	(Shintani *et al*., 2014)
*G.* sp. MAS1	Potential source of useful enzyme-encoding genes	NZ_AYSF01000001.1	(Siddiqui *et al*., 2014)
*G.* sp. WCH70	Not known	NC_012793.1	n. a.
*G.* sp. WSUCF1	Abel to grow on lignocellulosic substrates	NZ_ATCO01000001.1	(Bhalla *et al*., 2013)
*G.* sp. Y4.1MC1	Not known	NC_014650.1	n. a.
*G.* sp. Y412MC52	Not known	NC_014915.1	n. a.
*G.* sp. Y412MC61	Not known	NC_013411.1	n. a.
*G. stearothermophilus* ATCC 7953	Not known	JALS01000001.1	n. a.
*G. stearothermophilus* NUB3621	Genetically amenable host strain for metabolic engineering	AOTZ01000001.1	(Blanchard *et al*., 2014)
*G. thermocatenulatus* GS-1	Not known	JFHZ01000001.1	n. a.
*G. thermodenitrificans* NG80-2	Denitrification and degradation of long-chain alkanes, facilitating oil recovery in oil reservoirs	NC_009328.1	(Feng *et al*., 2007)
*G. thermodenitrificans* subsp. *thermodenitrificans* DSM 465	Comparative genomics between the alkane-utilizing NG80-2 and this strain which is unable to utilize alkanes	NZ_AYKT01000001.1	(Yao *et al*., 2013)
*G. thermoglucosidans* TNO-09.020	Contaminant in dairy-processing environment	NZ_CM001483.1	(Zhao *et al*., 2012)
*G. thermoglucosidasius* C56-YS93	Not known	NC_015660.1	n. a.
*G. thermoglucosidasius* NBRC 107763	Not known	BAWP01000001.1	n. a.
*G. thermoleovorans* B23 DNA	Alkane degrader with unidentified alkane monooxygenase	BATY01000001.1	(Boonmak *et al*., 2013)
*G. thermoleovorans* CCB_US3_UF5	Not known	NC_016593.1	(Muhd Sakaff *et al*., 2012)

Names are given as found in the GenBank sequence database. n.a., not available.

**Fig 3:**
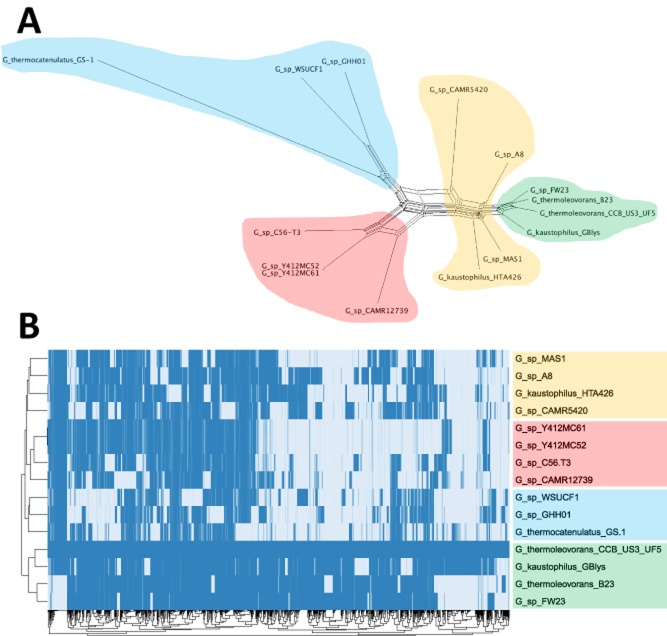
Relationships among sequenced genomes within the *G. kaustophilus* clade resolved using whole-genome sequence data. The phylogenetic network in panel A was based on a concatenation of 1722 variant single-nucleotide sites in 1 874 967 nucleotides of the core genome present in all 15 genomes. The network was generated using the neighbornetalgorithm (Bryant and Moulton, 2004) implemented in the splitstree software package (Huson, 1998). The heat-map in B indicates the presence (dark blue) and absence (light blue) of each of 931 non-core genes from the genome of *G. thermoleovorans* CCB US3 UF6 across the same 15 genomes appearing in A. The gene-content clusters are shaded in the same colours in both panels. The heat-map was rendered using Raivo Kolde's pheatmap package in R (R Development Core Team, R, 2013).

The phylogenetic group within *Geobacillus* most richly represented by genome sequences is the clade containing *G. thermoleovorans*, *G. kaustophilus* and *G. thermocatenulatus* (see the ‘kaustophilus clade’ in Fig. 2). Based solely of sequences of the *recN* phylogenetic marker, it is not possible to precisely resolve relationships among sequenced strains within this group (Fig. 2). However, the availability of complete genome sequence data enables phylogenetic analysis based on single-nucleotide variants over the entire core genome, offering much greater resolution (Fig. 3A). According to the core-genome-wide phylogenetic analysis, the two strains assigned as *G. kaustophilus* do not form a phylogenetically coherent monophyletic clade. On the other hand, the two strains of *G. thermoleovorans* are closely related and share 99.4% nucleotide sequence identity [based on mummer2 alignments (Delcher *et al*., 2002)]. Strain FW23 also appears to fall within this clade and, subject to phenotypic characterization, can probably be considered a member of this species too. *Geobacillus thermocatenulatus* GS-1 is much more divergent, sharing only 94% to 95% identity with the other strains in the clade, which is consistent with the *recN*-based analysis (Fig. 2). Strains Y412MC52 and YP412MC61 appear to be extremely closely related to each other, sharing 99.8% sequence identity and showing no detectable differences in gene content. Nucleotide sequence identities between clades are much lower; between *G. kaustophilus* and *G. thermoglucosidans*, there is approximately 84% identity.

The considerable amount of reticulation in the phylogenetic network (Fig. 3A) suggests significant horizontal genetic transfer within and among these species. This is further illustrated by the extent of variation in the variable component of the genome (Fig. 3B). Out of 3887 genes on the chromosome of *G. thermoleovorans* CCB US3 UF5, a total of 931 (approximately 24%) are variable (that is, they are absent from at least one of the other sequenced genomes). The global pattern of gene content (Fig. 3B) broadly reflects the phylogenetic relationships (Fig. 3A): according to gene content, the genomes fall into four main clusters, indicated by four different colours of shading in Fig. 3B, which correspond to four zones of the phylogenetic network, shaded with the same colours in Fig. 3A. However, there are numerous genes whose distribution across the genomes is incongruent with core-genome phylogeny, again suggesting extensive horizontal transfer.

## What benefits has the sequencing of *Geobacillus* genomes brought?

The availability of complete *Geobacillus* genome sequences has enabled or accelerated the discovery, cloning and exploitation of natural products. For example, the availability of the NG80-2 genome sequence (Feng *et al*., 2007) enabled the discovery of thermostable homologues of the lantibiotic nisin in *G. thermodenitrificans* (Begley *et al*., 2009; Garg *et al*., 2012), opening the possibility of replacing nisin as a food preservative and veterinary antibiotic with more-stable alternatives. Lantibiotics appear to be widely distributed among sequenced *Geobacillus* species. For example, the genome of *G. kaustophilus* HTA426 contains two lantibiotic-biosynthesis gene clusters (centred on the genes for YP_146139 and YP_146147) that are both conserved in the recently sequenced *Geobacillus* sp. CAMR12739. The NG80-2 genome sequence also enabled discovery of the first nitrous oxide reductase gene from a Gram-positive, and a novel thermophilic long-chain alkane monooxygenase (Feng *et al*., 2007). Furthermore, the genome sequence enabled proteomics-level confirmation of pathways for catabolism of long-chain alkanes (Feng *et al*., 2007) and aromatics (Li *et al*., 2012).

Many of the *Geobacillus* genome sequencing projects reported genes potentially encoding thermostable homologues of useful enzymes. In some cases, the genome sequences have been used to clone and express the genes of interest and characterize the enzyme for biotechnological potential. For example, the genome of *G. kaustophilus* HTA426 was recently mined for members of the glycoside hydrolase family 1, which have potential uses in synthesizing therapeutic oligosaccharides (Suzuki *et al*., 2013). The genome sequence of the alkane-utilizing *G. thermoleovorans* B23 (Boonmak *et al*., 2013) revealed a cluster of three long-chain alkane monooxygenase genes with homology to that of NG80-2 that showed activity *in vivo* when heterologously expressed in *Pseudomonas fluorescens* (Boonmak *et al*., 2014). Recently, a novel thermostable endo-xylanase was cloned and expressed from *Geobacillus* sp. WSUCF1 (Bhalla *et al*., 2014) following the sequencing of its genome (Bhalla *et al*., 2013).

Genome sequencing has revealed that interesting traits are often encoded on chromosomes rather than on the chromosome. For example, the biphenyl-degrading pathway of *Geobacillus* sp. JF8 (Mukerjee-Dhar *et al*., 2005; Shintani *et al*., 2014) and the long-chain alkane monooxygenase of *G. thermodenitrificans* NG80-2 (Feng *et al*., 2007) are both located on plasmids. The dynamic loss and gain of such mobile elements presumably explains, in part, the physiological differences between natural isolates of *Geobacillus* spp. and it also suggests that these bacteria might be engineered to express new traits by introduction of recombinant plasmids. Indeed, progress has been made in developing plasmid shuttle vectors for heterologous expression in *Geobacillus* spp. (Thompson *et al*., 2008; Bartosiak-Jentys *et al*., 2013).

The value of genome sequencing goes beyond cataloguing potentially useful enzymes, as exemplified by the recently published genomic study of strain NUB3621 (Blanchard *et al*., 2014). Some previous attempts to fully exploit the potential of *Geobacillus* strains as whole-cell catalysts have been frustrated by the paucity of genetic and genomic resources (my own PhD research project in the mid-1990s being a case in point; Studholme, 1998). However, strain NUB3621 is a promising laboratory workhorse strain. It is one of the few *Geobacillus* strains that has been shown to be readily transformable with plasmid DNA (Wu and Welker, 1989); protocols have been developed for genetic analysis (Chen *et al*., 1986) and a genetic map has been available for more than two decades (Vallier and Welker, 1990). Strain NUB3621 is a mutant derived from wild-type strain NUB36 that lacks its parent strain's restriction-modification system and this probably contributes to transformation efficiency. Incidentally, and consistent with this, we observed that transformation efficiency was significantly affected by the methylation status of the plasmid DNA (Thompson *et al*., 2008).

Being one of the most genetically amenable *Geobacillus* strains, NUB3621 was obviously a high priority for genome sequencing. But rather than simply announcing and describing its genome sequence, the authors went on to show how the genome sequence could be exploited to further develop the strain as a host for heterologous expression and metabolic engineering (Blanchard *et al*., 2014). Specifically, they used the genome sequence to clone two promoters and incorporated them into plasmid vectors: one for inducible gene expression and one constitutive. The authors also mention that they tried other promoters that did not work so well; presumably, the availability of the genome sequence allowed them to relatively quickly screen a number of candidates until they found the best ones. The combination of a genome sequence, allowing relatively facile construction of expression and/or knock-out constructs and a global view of metabolism, along with transformability and a wide range of growth temperatures [between 39 and 75°C (Wu and Welker, 1991)] make NUB3621 a strong candidate as the preferred thermophilic host for rationally designed metabolic engineering.

## What's next?

The availability of complete (or nearly complete) genome sequences for nearly 30 *Geobacillus* strains (Table 1) as well as large-scale proteomic data for at least one (Feng *et al*., 2007; Li *et al*., 2012) should certainly accelerate cloning, expression and characterization of novel thermostable and thermo-active enzymes, at least in an academic research context. However, there has been relatively little industrial uptake of enzymes from thermophiles, with much greater use of proteins originating from mesophiles but engineered for thermo-stability (Haki and Rakshit, 2003; Taylor *et al*., 2011). The convergence of genomic data and transformability, at least for strain NUB3621, should help to remove the barriers to greater exploitation of thermophiles. However, genome sequences are not yet publicly available for the handful of other readily transformable *Geobacillus* strains such as *G. thermodenitrificans* K1041 (Narumi *et al*., 1992), *G. stearothermophilus* IFO 12550 (Imanaka *et al*., 1982), NRRL 1174 (Liao *et al*., 1986) and *G. thermoglucosidasius* TN (Thompson *et al*., 2008). Furthermore, although it is possible to predict the metabolic networks of bacteria from complete genome sequence, there is a need for comprehensive testing of these predictions through metabolomics. Only then can we rationally design genetic interventions to predictably manipulate metabolism. And finally, palaeo-genomics of ancient *Geobacillus* spores, which may be viable after billions of years of dormancy, might shed light on population-genetics and evolutionary processes over timescales that we previously assumed to be intractable (Nicholson, 2003; Zeigler, 2005).
